# ASO Author Reflections: Enduring Impact of COVID-19 on Cancer Surgery Patients

**DOI:** 10.1245/s10434-024-15661-7

**Published:** 2024-06-21

**Authors:** Jae-Woo Ju, Ho-Jin Lee

**Affiliations:** 1 Department of Anesthesiology and Pain Medicine, Seoul National University Hospital, Seoul, Republic of Korea; 2 Department of Anesthesiology and Pain Medicine, Seoul National University College of Medicine, Seoul, Republic of Korea

## Past

The COVID-19 pandemic has left a lasting impact on cancer surgery patients, which may have been particularly profound. This pandemic has not only directly and severely affected the prognosis of cancer surgery patients but also has led to a widespread depletion of medical resources, preventing cancer patients from receiving timely treatment.^1^ Early in the pandemic, a landmark study by the COVIDSurg and GlobalSurg Collaboratives found a significant correlation between recent COVID-19 infection within 7 weeks and increased postoperative mortality, which influenced decisions on elective surgeries.^2^ However, determining the optimal timing of surgery in cancer patients with preoperative COVID-19 infection remains a critical issue, as delayed cancer surgery could lead to a worsening prognosis because of cancer progression.

## Present

Fortunately, the impact of preoperative COVID-19 on postoperative outcomes has evolved because of the reduced virulence and vaccine availability of the virus. In our study, which utilized nationwide cohort data from South Korea in 2022, we found that preoperative COVID-19 infection in cancer surgery patients remained significantly associated with early postoperative mortality.^3^ However, this association was significant only for infections occurring within 2 weeks before surgery. The nonsignificant association between surgeries performed more than 2 weeks after COVID-19 infection and early postoperative mortality was more robust than the significant association found with surgeries conducted within 2 weeks of COVID-19 infection. This result aligns with the revised guidelines in 2023 regarding the recommended timing for elective surgery in patients with COVID-19 infection.^4^ Additionally, our study revealed that full vaccination against COVID-19 was associated with reduced postoperative mortality, highlighting the importance of vaccination in mitigating the risks for cancer surgery patients during the pandemic. Compared with our previous study using nationwide cohort data from South Korea in 2021, we observed a weaker impact of preoperative COVID-19 on early postoperative mortality.^5^

## Future

Although the COVID-19 pandemic has transitioned to an endemic phase because of the tireless efforts of many, its risk cannot be ignored. Further research is needed to understand the impact of COVID-19 on cancer surgery patients in this endemic era. Additionally, our study found a positive effect of full vaccination against COVID-19 before surgery, but most individuals have not received additional booster shots since then, necessitating a reassessment of the effectiveness of preoperative vaccination. These efforts will help to determine the optimal timing for cancer surgery in patients during the ongoing COVID-19 endemic era, ultimately improving surgical outcomes and overall prognosis.
